# APE2: catalytic function and synthetic lethality draw attention as a cancer therapy target

**DOI:** 10.1093/narcan/zcad006

**Published:** 2023-02-06

**Authors:** Anne McMahon, Jianjun Zhao, Shan Yan

**Affiliations:** Department of Biological Sciences, University of North Carolina at Charlotte, Charlotte, NC 28223, USA; Department of Cancer Biology, Lerner Research Institute, Cleveland Clinic, Cleveland, OH 44195, USA; Department of Biological Sciences, University of North Carolina at Charlotte, Charlotte, NC 28223, USA; School of Data Science, University of North Carolina at Charlotte, Charlotte, NC 28223, USA; Center for Biomedical Engineering and Science, University of North Carolina at Charlotte, Charlotte, NC 28223, USA

## Abstract

AP endonuclease 2 (APE2, APEX2 or APN2) is an emerging critical protein involved in genome and epigenome integrity. Whereas its catalytic function as a nuclease in DNA repair is widely accepted, recent studies have elucidated the function and mechanism of APE2 in the immune response and DNA damage response. Several genome-wide screens have identified APE2 as a synthetic lethal target for deficiencies of BRCA1, BRCA2 or TDP1 in cancer cells. Due to its overexpression in several cancer types, APE2 is proposed as an oncogene and could serve as prognostic marker of overall survival of cancer treatment. However, it remains to be discovered whether and how APE2 catalytic function and synthetic lethality can be modulated and manipulated as a cancer therapy target. In this review, we provide a current understanding of alterations and expression of APE2 in cancer, the function of APE2 in the immune response, and mechanisms of APE2 in ATR/Chk1 DNA damage response. We also summarize the role of APE2 in DNA repair pathways in the removal of heterogenous and complexed 3’-termini and MMEJ. Finally, we provide an updated perspective on how APE2 may be targeted for cancer therapy and future directions of APE2 studies in cancer biology.

## Graphical Abstract

**Graphical Abstract:**
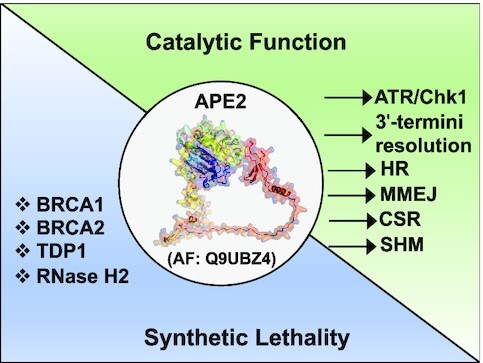
Catalytic function and synthetic lethality of APE2 (Alphafold structure of human APE2: Q9UBZ4). HR: homologous recombination; MMEJ: microhomology mediated end joining; CSR: class switch recombination; SHM: somatic hypermutation.

## INTRODUCTION

AP endonuclease 2 (APE2, also known as APEX2 and APN2) is an emerging protein of interest in the context of cancer and genome stability (1–3). The gene encoding human APE2 (Ensemble ID: ENSG00000169188) is located at the cytogenetic band Xp11.21 on the X chromosome between the coding regions for 6-phosphofructo-2-kinase/fructose-2,6-bisphosphatase 1 (PFKFB1) and 5'-aminolevulinate synthase 2 (ALAS2), which are involved in glycolysis and heme biosynthesis, respectively (2,4). Human APE2 is localized primarily to the nucleus and to a lesser degree the mitochondria (5,6). APE2 was first identified in yeast (APN2/ETH1 in yeast) and was initially characterized as a redundant and less efficient homologous endonuclease to AP endonuclease 1 (APE1) in *Saccharomyces cerevisiae* (4,7,8). APE2 differs from APE1 through possession of a unique C terminus region containing a conserved Zf-GRF motif as well as a PCNA-interacting protein (PIP) box located between AA390–397 in human APE2 (1,9). Subtle differences are also present in the active site exonuclease/endonuclease/phosphatase (EEP) domain that have dramatic effects on the catalytic activities possessed by these two paralogs (10). APE2-knockout (KO) mice are not embryonically lethal like APE1-KO mice (11,12), however in p53-proficient human epithelial cells APE2 loss is lethal, suggesting increased relevance of human APE2 as compared to its mouse counterpart (10).

APE2’s ‘endonuclease’ designation is somewhat of a misnomer. Strong evidence from multiple groups suggests that APE2 possesses strong 3'-5' exonuclease activity and 3' phosphodiesterase activity but relatively weak endonuclease activity *in vitro* (9,13–16). Using templated DNA constructs, APE2 has been shown to favor excision of mismatched base pairs as well as preferentially removing dA rather than dC in the case of inappropriate base pairing to 8-oxoguanine (8-oxoG) during DNA replication (13,14). APE2’s 3'-5' exonuclease activity generates large tracks of single-strand DNA (ssDNA) for the recruitment of RPA and ATR activation complex onto damage sites, leading to the activation of ATR/Chk1 DNA damage response (DDR) pathway in *Xenopus* egg extracts system (3,16,17). siRNA-mediated knockout of APE2 has been shown to eliminate ATR/Chk1 DDR in response to genotoxic stress in pancreatic cancer cells (16,18), indicating that APE2 may be highly regulated and differentially activated only under certain conditions to control ATR/Chk1 DDR.

Besides its exonuclease activity, APE2 has also been shown to efficiently remove 3'-blocks arising from phosphoglycolate adducts, ribonucleotide mis-incorporation, and Topoisomerase 1 cleavage complex (Top1cc) (10,13,14,19,20). Dirty 3'-end processing is essential for cell viability because polymerase extension requires an accessible 3'-OH for elongation (21). Replication fork collapse at inappropriately repaired AP sites or 3'-adducts lead to highly mutagenic double strand breaks which can result in chromosomal translocations and aneuploidy (22,23). Interestingly APE2 was recently shown to be synthetically lethal in concurrent knockout with TDP1, a 3'-adduct end processing enzyme (24). APE2’s rising significance is also due to encouraging work highlighting its synthetic lethality in homologous recombination (HR)-deficient cells (10,25,26). APE2 presents a promising candidate drug target in the case of PARP inhibitor and Pol Theta inhibitor resistant BRCA1/BRCA2 deficient tumors. To date, Celastrol has been characterized as one molecular inhibitor of APE2 but not APE1 (18). In this review, we will discuss recent findings on the significance of APE2 in the context of cancer including synthetic lethality of APE2 in HR-deficient cells (10,25,26), APE2 overexpression trends in cancer types (27), APE2’s role in ATR-Chk1 DDR (16,18), and the newly proposed role of APE2 in the highly mutagenic microhomology mediated end joining (MMEJ) (26). In addition, we will discuss the viability of APE2 as a drug target for cancer treatment.

## ALTERATIONS AND EXPRESSION OF APE2 IN CANCER

Bioinformatic analysis of APE2 in multiple types of cancer has shown that APE2 genomic alterations occur at a ∼17% frequency across 14 different cancer types (*n* = 21,769) (27). Additionally, APE2 mRNA expression was significantly upregulated in tumor tissues such as kidney, breast, lung, liver and uterine cancers compared with non-malignant tissues (27,28). A previous study has also shown that APE2 is upregulated at protein and mRNA levels in multiple myeloma (MM) cells compared to normal cells (29).

**Figure 1.**
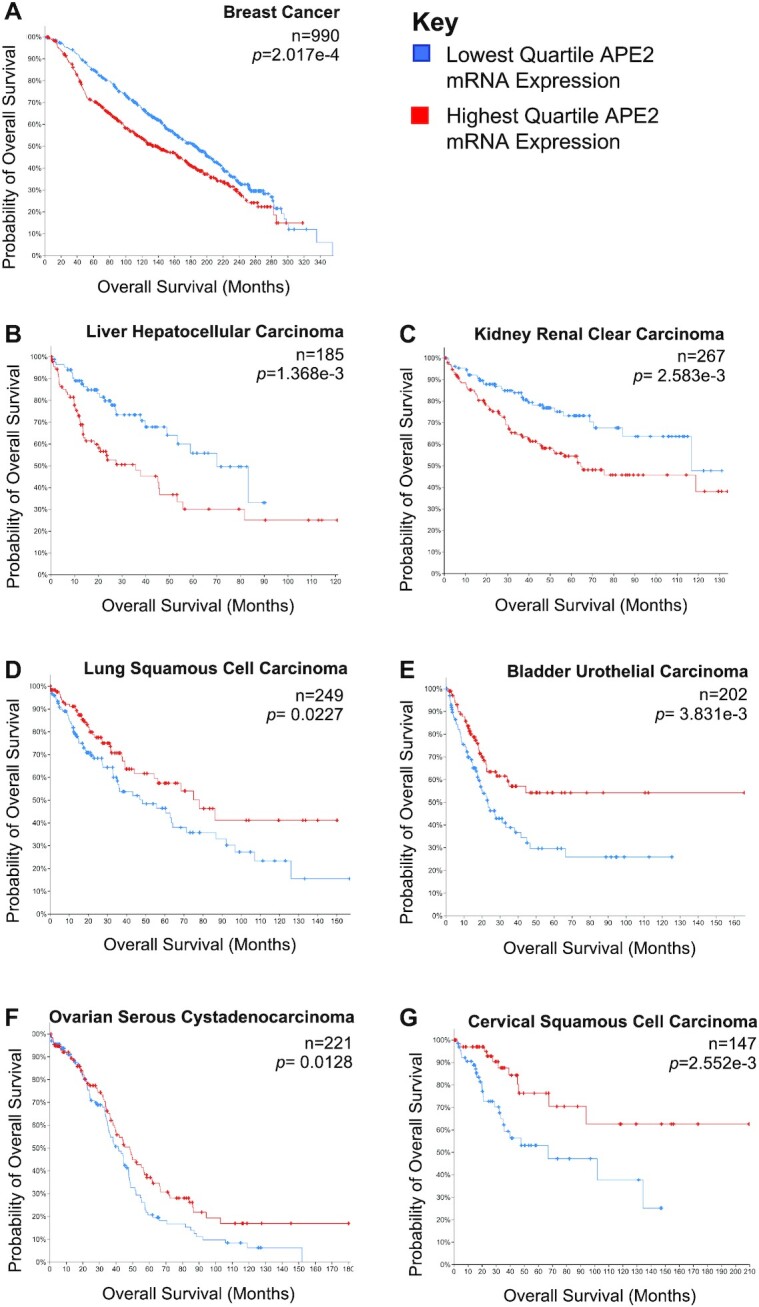
Overall survival of patients with high (red) and low (blue) APE2 mRNA expression. Comparison uses the highest and lowest quartile of APE2 mRNA expression from the following studies in cBioPortal: Breast Cancer (METABRIC, Nature 2012 & Nat Commun 2016), Liver Hepatocellular Carcinoma (TCGA, Firehose Legacy), Kidney Renal Clear Carcinoma (TCGA, Firehose Legacy), Lung Squamous Cell Carcinoma (TCGA, Firehose Legacy), Bladder Urothelial Carcinoma (TCGA, Firehose Legacy), Ovarian Serous Cystadenocarcinoma (TCGA, Firehose Legacy), Cervical Squamous Cell Carcinoma (TCGA, PanCancer Atlas). P-value was calculated using log-rank test. Graphs were generated by cBioPortal. *n* represents total number of patients compared.

Furthermore, APE2 overexpression is not only found in liver cancer patients, but is also associated with worse overall survival (28). APE2 is thus proposed as an oncogene in liver cancer. cBioportal analysis of cancer patients (30) further shows that high APE2 mRNA expression is associated with worse overall survival in breast cancer, liver hepatocellular carcinoma, and kidney renal clear carcinoma (Figure 1A–C). However, high APE2 mRNA expression is associated with better overall survival in lung squamous cell carcinoma, bladder urothelial carcinoma, ovarian serous cystadenocarcinoma, and cervical squamous cell carcinoma patients (Figure 1D–G). These data suggest that APE2 may play differential roles in different cancer types as an oncogene or tumor suppressor gene. Although future mechanistic studies are needed to understand the differential effects of APE2 expression and activity in multiple cancer types, this putative cancer-specific difference remains unexplored. Current evidence shows that APE2 can activate ATR/Chk1 DDR (16–18), which promotes faithful replication and acts antithetically to the development of cancer and thereby serves a role equivalent or similar to tumor suppressor. On the other hand, overexpressed APE2 in germinal center B cells promotes error-prone repair or mutagenesis, and APE2 expression is positively associated with cell cycle protein CCNB1 and pro-oncogenic MYC in liver cells, suggesting a potential role of oncogene (28,31). More conclusive evidence illuminating APE2’s role in MMEJ may also give some idea as to why APE2 expression leads to variable effects in different cancer types as MMEJ’s role in clinical outcomes is poorly understood. Additional mutations unique to cancer types likely dictate APE2’s efficacy as a tumor suppressor or oncogene.

## FUNCTION OF APE2 IN IMMUNE RESPONSE

**Figure 2.**
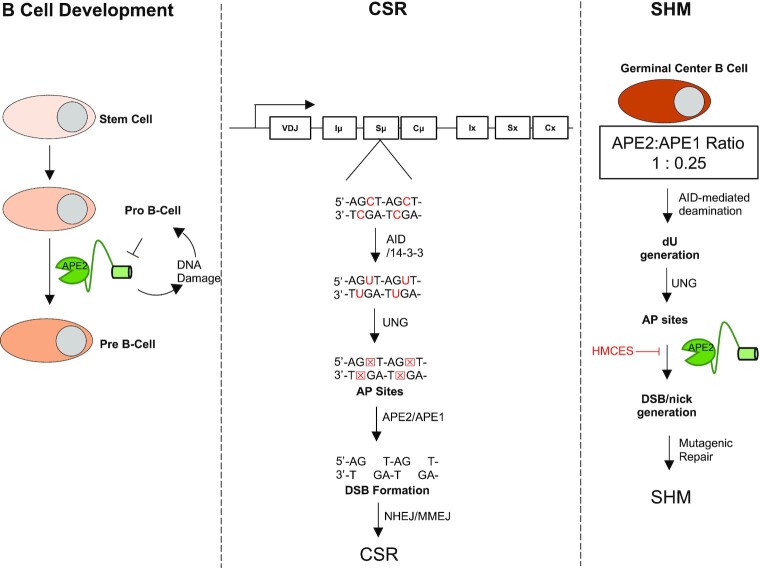
Roles of APE2 in B cell development, class switch recombination (CSR), and somatic hypermutation (SHM).

While APE1-KO mice are embryonically lethal (12), APE2-KO mice are viable (11). APE2-KO male mice (*APE2^−^^/Y^*) are slightly smaller in size and have defects in lymphopoiesis (11). APE2-KO mice do not show obvious DNA repair defects but do show ∼50% reduction in B and T cells (11). In germinal center B cells, APE1 expression is dramatically downregulated and APE2 expression is upregulated, effectively replacing APE1 as the dominantly expressed endonuclease (31). Several previous studies have shown that APE2 is critical for proper lymphocyte development, class switch recombination (CSR) and somatic hypermutation (SHM) (Figure 2) (31–35). Specifically, *APE2^−^^/Y^* mice are inhibited at the pro-B to pre-B cell transition (35). This development inhibition was further exaggerated in *APE1*^−/+^ cross with *APE2^−^^/Y^* however no phenotype is observed in *APE1*^−/+^ mice alone suggesting that APE2 plays a major role in B cell development (Left panel, Figure 2) (35). In this case, the authors propose that APE2’s role in the repair of Activation Induced Cytidine Deaminase (AID)-independent oxidative damage generated by proliferating cells is critical for pro-B to pre-B cell transition (34,35).

CSR requires proximal SSB generation in the switch region of germline DNA (36). SSB targeting is performed by AID which deaminates deoxyribose cytosine to deoxyribose uracil (36,37). This is recognized and excised by uracil–DNA glycosylase (UNG), forming an apurinic/apyrimidinic (AP) site. APE1 and APE2 act downstream of UNG to convert the AP site into a SSB and a DSB is generated when two SSBs are formed on opposite strands in proximity (32). CSR decreases 65% of wild type in *APE2^−^^/Y^* mice after stimulation (33). Surprisingly, *APE2^−^^/Y^* mice do not show further reduction of CSR after *APE1^−^^/+^*, indicating that APE2 activity is more important than APE1 for DSB formation in CSR (Middle panel, Figure 2). Using ligation mediated PCR to detect DSB formation in B cell switch regions, *APE1^−^^/+^*/*APE2^−^^/Y^* mice B cells showed reduced DSB formation and loss of AID-targeting hotspot compared to WT (Figure 2) (32,33). This evidence taken together highlights the importance of APE2 in the normal function of B cells. With recent developments suggesting that APE2 plays a critical role in MMEJ and the ability of MMEJ to repair DSB and promote CSR in the absence of NHEJ, it will be interesting to see if this newly proposed function of APE2 factors into CSR and overall lymphocyte function (26,38). The closely linked relationship between APE2 and lymphocyte cell development is noteworthy because of the germline editing role of APE2 in these cells.

Compared with APE1, APE2 is upregulated in germinal center B cells (∼4-fold) and is critical for SHM, increasing the frequency of mutation and increasing the rate of A:T transversions (right panel, Figure 2) (31,39). Proliferation of germinal center B cells is greatly reduced upon APE2-KO (31). APE2’s role in SHM may be due to its generation of long tracks of ssDNA which results in the recruitment of error prone translesion synthesis polymerases such as Pol η and Rev1. Surprisingly, knockout of UNG in combination with APE2 knockout caused an additional and dramatic reduction in SHM, indicating that APE2 acts in a distinct pathway separate from the canonical AID, UNG, and APE1 mutagenesis pathway (31). A recent report suggests that HMCES protects AP sites on ssDNA from APE2-mediated cleavage into DSB formation in SHM (Right panel, Figure 2) (40). In addition, APE2 might be able to incise at dU in a similar manner to APE1 (41), although a definitive explanation for this observation remains unclear.

APE2 is required for both DNA repair in the case of lymphocyte development, and intentional DNA damage, in the case of SHM and CSR. APE2 in lymphocytes is therefor likely to be tightly regulated through abundance, post translational modifications (PTMs), and/or subcellular localization. Dysfunction of APE2 in lymphocytes is a likely cause or consequence of lymphoma. In accordance, APE2 protein and mRNA respectively are shown to be greatly upregulated in multiple myeloma, which arises from plasma cells upstream of B and T cells in lymphocyte development (29). The relevance of APE2-KO in B cell development is well studied, however the effect of APE2 overexpression in lymphocytes warrants further studies.

## MECHANISM OF APE2 IN ATR-CHK1 DNA DAMAGE RESPONSE

**Figure 3.**
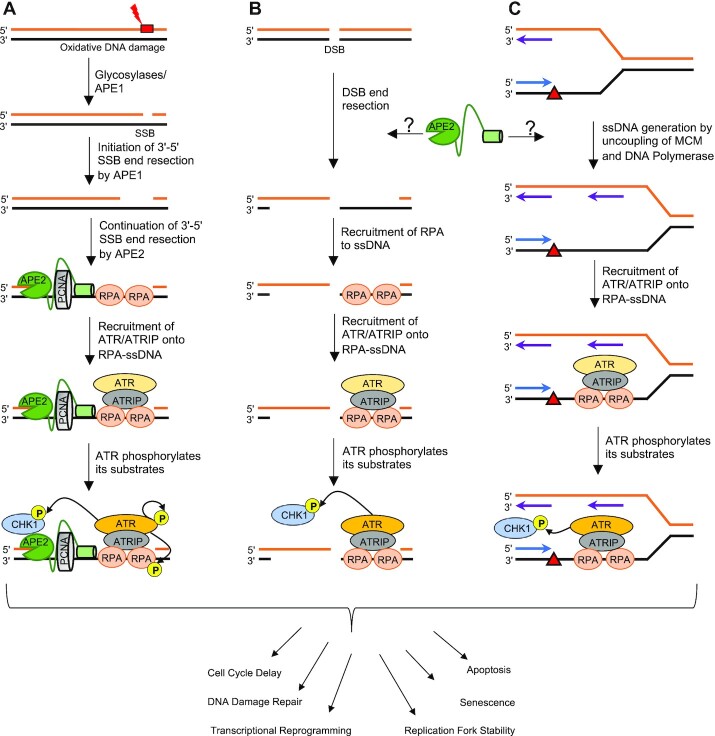
Role and mechanism of APE2 in the ATR/Chk1 DDR pathway. (**A**) Oxidative DNA damage and DNA single-strand breaks (SSBs). (**B**) DNA double-strand breaks (DSBs). (**C**) DNA replication stress.

Phosphatidylinositol 3-kinase-related kinase proteins (PIKKs) including Ataxia Telangiectasia mutated (ATM), ATM and Rad3-related protein (ATR) and DNA-dependent protein kinase catalytic subunit (DNA-PKcs) are serine/threonine kinases largely responsible for the coordination of the DDR (42,43). Originally, researchers thought PIKK proteins directed one and only one DDR process; ATM directs DSB repair via HR, DNA-PKcs directs DSB repair via non-homologous end joining (NHEJ), and ATR directs the resolution of stalled DNA replication stress and SSB repair at replication forks. However, these DDR and DNA repair pathway crosstalk and regulate each in a context-dependent manner and a more dynamic model has taken its place (43,44). For example, we now know that ATM can directly phosphorylate DNA-PKcs to abort NHEJ (45) and that ATR signaling can be activated from resected tracts of ssDNA generated by Exo1 from DSB ends (42). Here, we summarize what we know about the role and mechanisms of APE2 in ATR/Chk1 DDR pathways (Figure 3).

The first line of evidence of APE2 in ATR/Chk1 DDR signaling was characterized in the context of oxidative stress in *Xenopus laevis* low-speed supernatant (LSS) system (Figure 3A) (9,17,46). Consistent with the theory that ATR is activated by RPA-coated ssDNA (47), one role of APE2 in ATR activation after oxidative stress is generating long tracks of ssDNA on chromatin DNA for RPA binding and subsequent recruitment of ATR/ATRIP, Rad9-Rad1-Hus1 complex, and TopBP1 (Figure 3A) (16–18). Activated ATR phosphorylates many substrates including Chk1, RPA32, H2AX and p53 (48,49). APE2 depletion in the LSS system attenuates ATR/Chk1 signaling in response to oxidative stress (17). Rescue of ATR/Chk1 signaling was seen with the reintroduction of wild type *Xenopus laevis* APE2 (xlAPE2) but not nuclease-deficient mutants suggesting that APE2 nuclease activity is critical for the activation of ATR/Chk1 signaling. Additionally, xlAPE2 was shown to interact directly with Chk1 via Ser86 which is conserved in humans (Ser100) and mice (Ser99). Mutating Ser86 in xlAPE2 attenuated Chk1 phosphorylation at S344 (S345 in humans) but not RPA phosphorylation by ATR. APE2 therefore likely acts not only to generate large tracks of ssDNA for RPA binding and ATR recruitment, but also directly recruits Chk1 via protein-protein interaction (Figure 3A) (17). Of note, the Zf-GRF motif of xlAPE2 was crystalized and shown to possess ssDNA binding capability that proved essential for APE2 3'-5' resection in response to oxidative stress (9).

The second line of evidence of APE2 in ATR/Chk1 DDR pathway was demonstrated in response to a defined plasmid-based SSB structure in *Xenopus laevis* high-speed supernatant (HSS) system (16,46). Whereas the ATR/Chk1 DDR activation by oxidative stress in the LSS system is dependent on DNA replication (9,17), the ATR/Chk1 DDR signaling induced by defined SSB structures in the HSS system is independent of DNA replication (16). APE2’s exonuclease activity was found essential for the 3'-5' SSB end resection for ATR activation in the HSS system (Figure 3A) (16). Futher mechanistic studies elucidated a downstream function of APE2 to continue 3'-5' SSB end from a small gapped structure initiated by APE1 (Figure 3A) (50). It appears that sequential and coordinated actions by APE1 and APE2 promote the ATR/Chk1 DDR pathway in a non-replicating context (Figure 3A) (3,16,50).

The third line of evidence of APE2’s role in ATR DDR was shown in pancreatic cancer cells. siRNA-mediated knockdown of APE2 (APE2-KD) attenuates ATR/Chk1 signaling in response to oxidative stress, DNA replicative stress, and DSBs (18). ATR/Chk1 signaling was restored however upon reintroduction of siRNA resistant xlAPE2 in APE2-KD cells. Importantly, the ability of xlAPE2 to activate ATR/Chk1 signaling is preserved in mammalian cells, adding significance to prior work in *Xenopus* models (9,16,17). This evidence suggests that APE2 regulates ATR signaling through its ability to generate RPA-ssDNA from SSBs derived from oxidative DNA damage and other damage types (Figure 3A). Therefore, APE2 may be critical as a first responder to increasing SSB loads in a cell to promote ATR activation and thus cell cycle delay, DNA repair, and, if necessary, apoptosis. Oxidative stress is known to activate inflammatory pathways such as NF-kB and p53 (Figure 3) (51,52). Cancer initiation and transformation has been closely linked with oxidative stress and inflammation, thus the role of APE2 in ATR activation may be a critical determinant in the prevention of tumor cell formation. More evidence is needed in other cell types to make broader conclusions about APE2 in ATR signaling. However, the underlying mechanism of APE2 in ATR DDR induced by DSBs and DNA replication stress warrants further investigation. For example, does APE2 affect the 5'-3' DSB end end resection that is needed for ATM to ATR transition in DSB signaling (Figure 3B) (53)? Does APE2 regulate the uncoupling of MCM helicase and DNA polymerase and/or the recruitment of ATR/ATRIP complex onto RPA-coated ssDNA at stalled DNA replication forks (Figure 3C) (54)? In addition, ATR signaling occurs during DNA replication without exogenous stressors to ensure high fidelity during DNA replication (55). Dysfunction of ATR signaling during DNA replication may result in inappropriate cell cycling and improper separation of chromosomes among dividing cells leading to aneuploidy (56). The role of APE2 during DNA replication and ATR signaling by endogenous DNA damage remains to be fully investigated, however two key studies give us some clue as to APE2’s role in ATR signaling during DNA replication. First, APE2 expression at both mRNA and protein is upregulated during early S phase in mouse BALB/c 3T3 cell (57). Second, upon replication fork stalling, Dungrawala et al. isolated the replisome and identified APE2 as being highly enriched at DNA replication forks (58). Taken together, these lines of evidence point at a role of APE2 in ATR signaling during DNA replication, although the underlying mechanism remains to be understood.

A critical regulator of APE2 function is PCNA, which interacts and regulates APE2 in ATR DDR pathways. APE2 has been shown to colocalize with PCNA under oxidative stress but not alkylation DNA damage (14). While APE2 does not require PCNA interaction for *in vitro* 3'-5' end resection, the addition of PCNA greatly enhances the exonuclease activity and the phosphodiesterase activity of APE2 (14). APE2 interaction with PCNA occurs via two modes. The first mode of APE2 interaction with PCNA occurs via the conserved PIP Box on APE2 and the Interdomain Connecter Loop (IDCL) of PCNA. This mode of interaction is conserved across *Xenopus*, yeast, and humans (14,15,17). The second mode occurs via the APE2’s Zf-GRF and the C-terminal Motif (CTM) of PCNA (16). Both modes of APE2-PCNA interaction are required for the activation of ATR DDR in *Xenopus* LSS/HSS systems (16,17). During investigations on the cause of synthetic lethality (SL) of APE2 in BRCA2^−/−^ cell lines, Mengwasser et al. found that wild type but not R339A mutant of APE2 could rescue the SL phenotype (25), in which R339A mutant was claimed to ablate APE2-PCNA interaction. However, the R339A mutation falls outside of the canonical PIP box (AA390-397) and the non-canonical PCNA-interaction via Zf-GRF motif of APE2 (1,14,16). In a follow up study, Alvarez-Quilon *et al.* found that while catalytically dead APE2 (E48Q/D197N) did not rescue SL phenotype in BRCA1^−/−^ cells, the PIP box and Zf-GRF deletion mutant of APE2 did rescue viability in these cells (10). Futures studies are needed to further validate whether the APE2-PCNA interaction is critical for the SL of APE2 in BRCA1^−/−^ or BRCA2^−/−^ cells.

## ROLE OF APE2 IN DNA REPAIR

### APE2 resolves endogenous DNA 3'-termini

**Figure 4.**
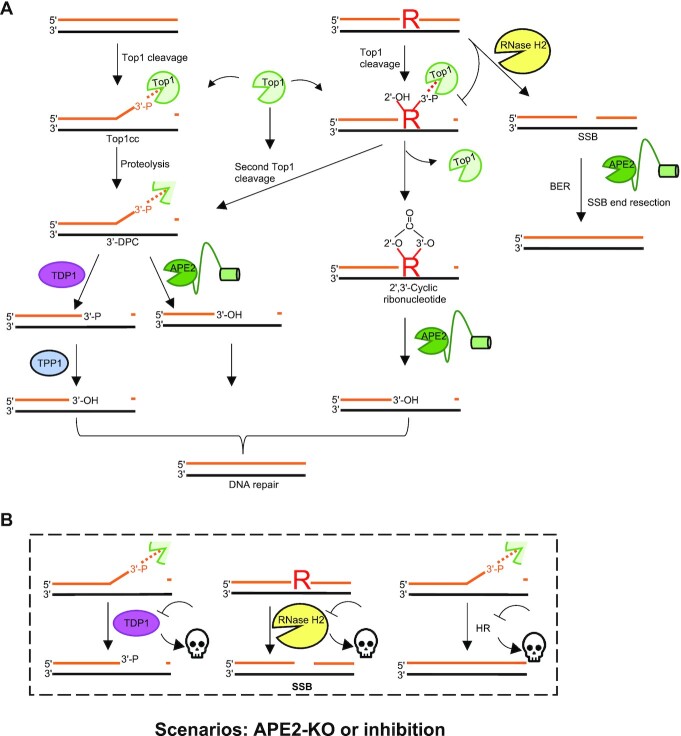
Role of APE2 in the removal of heterogenous 3’-termini. (**A**) Mechanisms of APE2 3’ end processing. R represents ribonucleotide incorporated into DNA. Dashed lines represent DNA-protein adducts. (**B**) Synthetic lethality in the case of APE2 knockout or inhibition. Skull represents loss of cell viability. KO = knockout.

Yeast APN2 and human APE2 have both been shown to resolve complex 3'-termini templated DNA constructs *in vitro* (10,20). Complex 3'-termini lack an accessible OH group for polymerase extension and can arise from multiple sources including DNA-protein crosslinking commonly observed during Topoisomerase I (Top1) catalytic cycle. 3'-termini blocks occur at Top1 cleavage intermediates which are especially prevalent at sites of ribonucleotide misincorporation as Top1 cleavage of ribonucleotides leads to 2',3' cyclic ribonucleotide 3'-blocks (59–61). 3'-blocks can also occur due to cleavage of AP sites by DNA lyase and can be exogenously introduced by the addition of PARPi which result in PARP trapping (62). Proteolytic cleavage of Top1 cleavage complex (Top1cc) is further processed or removed by TDP1 or APE2 *in vitro*, with TDP1 products harboring 3'-P and APE2 products harboring 3'-OH (Left panel, Figure 4A) (10). Therefore, TDP1 and APE2 likely have distinct cellular contexts. In support of this, a recent study observed a synthetic lethal phenotype of APE2-KO in TDP1-deficient background and that these cells had compounding sensitivity to CPT and MMS (24). APE2’s cellular context for processing 3'-ends is likely linked with repair of misincorporated ribonucleotides into DNA. Loss of fitness in cells lacking APE2 was observed after depletion of *RNASEH2A* and *RNASEH2B* respectively (10). The loss of *RNASEH2A* and *RNASEH2B* results in attenuation of the cells ability to excise misincorporated ribonucleotides from DNA. Loss of fitness in cells lacking both APE2 and *RNASEH2A* or *RNASEH2B* is likely due to an overwhelming number of unprocessed Top1cc formed at misincorporated ribonucleotides. Due to its function in the resolution of 3'-termini blocks, APE2 is a SL target of TDP1- and RNase H2-deficiency in HR-proficient tumors (Figure 4B).

APE2 was identified as a SL target of BRCA2-deficient colorectal adenocarcinoma DLD-1 and ovarian adenocarcinoma PEO1 cells using both shRNA and CRISPR based screening methods (25). The authors proposed that the SL is due to a role of APE2 in processing of AP sites at DNA replication forks (25). It was reported that Chk1 and RPA32 phosphorylation was upregulated after APE2 depletion in BRCA2-mutant but not BRCA2-WT cells (25). An additional CRISPR screen by an independent group came to the same conclusion that APE2 is SL target of BRCA1/2-deficient cells (10). An alternative explanation is offered by Alvarez-Quilon et al. that PARPi is synthetically lethal to BRCA1- and BRCA2-deficient cells because of PARP trapping which generates 3'-blocked ends, and that PARP trapping often occurs at ribonucleotides inappropriately incorporated into DNA (63,64). In yeast, APN2 has been shown to resolve 3'-blocked ends and repair Top1-generated damage at ribonucleoside monophosphate sites (rNMP) (Middle panel, Figure 4A) (20). In mammalian cells, Alvarez-Quilon et al. generated two mutants (Y396A/F397A and PIP-deletion) of APE2 which they show to be unable to interact with PCNA in a pulldown assay (10), and found that PCNA interaction was dispensable in SL rescue experiments, suggesting an alternative, PCNA independent, function of APE2 is responsible for SL phenotype (10,25). Of note, Alvarez-Quilon et al. denote the PIP box motif of APE2 as AA395–402; however, the human APE2 PIP box motif is located between AA390–397 (1,5,10). Nonetheless, the critical role of APE2 in the resolution of 3'-termini blocks may also be relevant for the SL phenotype observed in HR-deficient tumors (Figure 4B).

### APE2 in MMEJ

MMEJ is an error prone form of DSB repair that canonically involves PARP1, XRCC1, Pol Theta, FEN1 and Lig3 (65–68). MMEJ utilizes microhomologies of ∼3–20nt around the site of the DSB to reanneal the break (38). This form of DSB repair is supplemental to NHEJ and is known to be responsible for large deletions and insertions in the genome. Knockout of MMEJ factor is SL to cells lacking HR machinery for an unknown reason, although the running theory is that MMEJ repair can act as a compensatory mechanism for DSB repair in the absence of HR, especially in the repair of DSBs arising from fork collapse (69–71). MMEJ is known to be upregulated in cancer and many cancer cells become reliant on this mechanism of DSB repair while normal cells are not. MMEJ is also mutagenic in nature and causes of genomic deletions and insertions (72–74). Thus, targeting MMEJ effector proteins may prove to be an effective strategy in cancer treatment and possibly reduce cancer heterogeneity by limiting the efficacy of this highly mutagenic repair pathway. In agreement with this, Pol Theta inhibitors have been/are being tested for their efficacy as chemotherapy (75).

**Figure 5.**
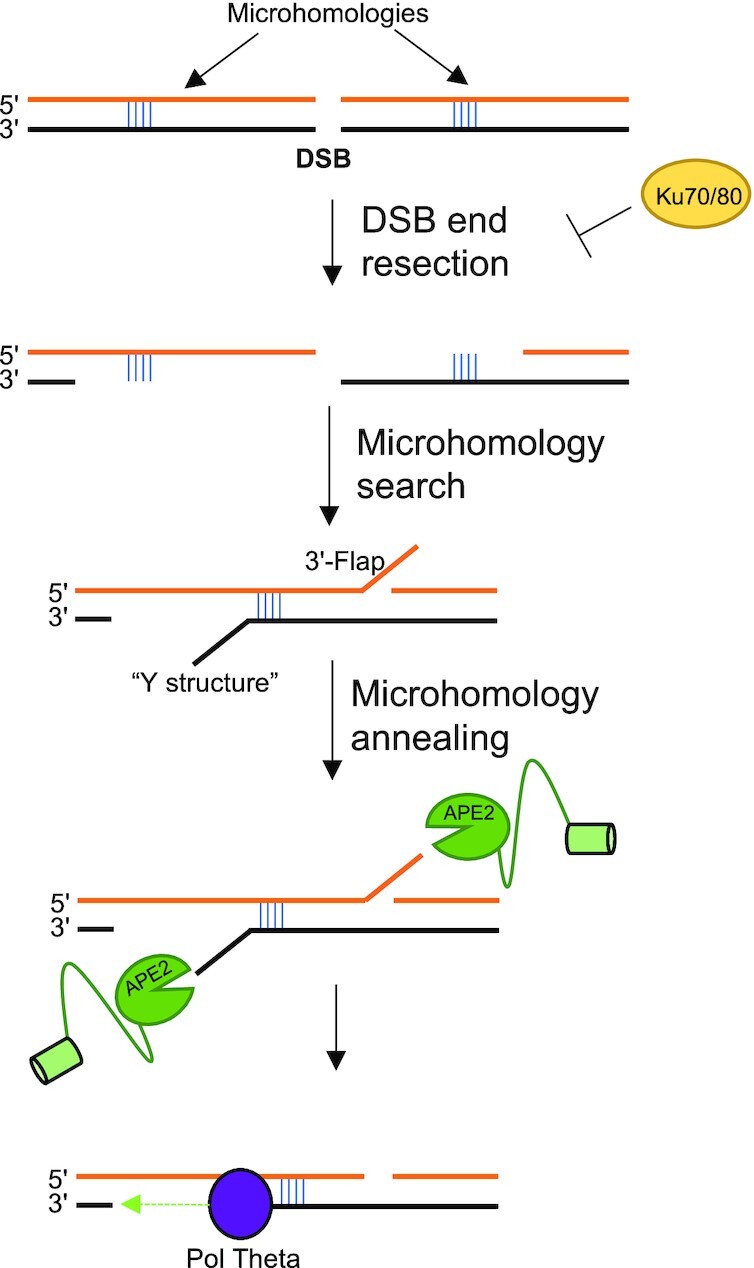
Tentative role of APE2 in 3’-5’ exonuclease processing of 3’-Flaps and Y-structures generated during MMEJ.

A critical role of APE2 in MMEJ repair of DSB was recently proposed (26). It was shown that telomere fusion due to MMEJ was reduced from 20% of cells to <5% of cells after APE2-KO, which was a similar reduction to that of Pol Theta-KO (26). Additionally, a fluorescent MMEJ reporter construct showed that the amount of MMEJ repair after ISce1-induced DSB was greatly reduced after APE2-KO. APE2’s SL phenotype with BRCA1-deficient cells could be due to its role in MMEJ especially considering APE2’s previously uncharacterized role in the removal of 3’-Flap and Y-structures generated in MMEJ (Figure 5) (26). The authors further characterized that deletion of the Zf-GRF but not PIP box had significant reduction on its ability to restore MMEJ in rescue experiments in comparison with WT APE2. Previous work has shown that APE2 and PCNA interact via both the PIP box and Zf-GRF motif (14,16), thus the authors claim that APE2’s role in MMEJ is PCNA-independent remains unproven. Interestingly, two conserved regions, denoted CR1 (AA324–342) and CR2 (AA447–463), were identified to delay or completely ablate APE2’s ability to respond to irradiation-induced DNA damage foci in live cell imaging. Mutation in CR1 impaired APE2’s ability to localize to the nucleus, suggesting that CR1 contains a nuclear localization signal but CR2’s function remains to be identified. In total, APE2 is likely involved in MMEJ although the full extent and consequence of APE2’s involvement is only now starting to be explored. Whether or not APE2’s SL phenotype with BRCA1-deficient cells is due to its involvement in MMEJ or in 3'-block removal remains unclear, however APE2’s SL phenotype with *RNASEH2* fails to be explained by the MMEJ ablation model.

## APE2 AS A TARGET FOR CHEMOTHERAPY

**Figure 6.**
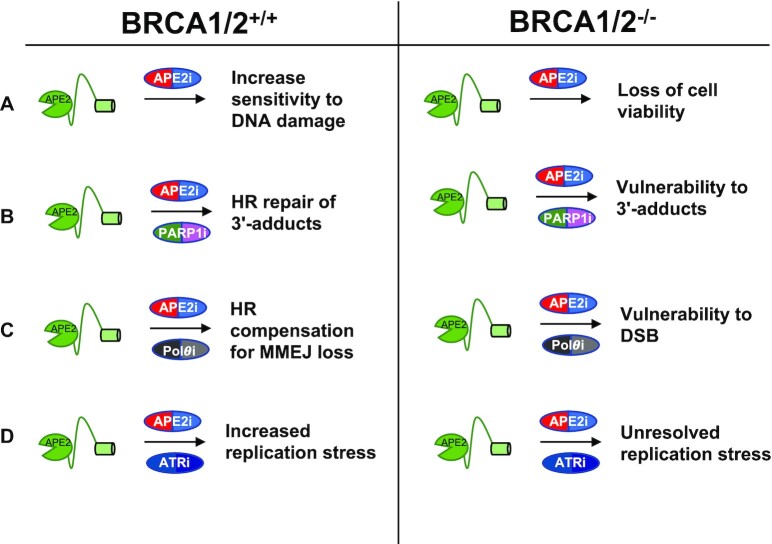
Effects and consequences of APE2 as a drug target for therapies in BRCA1/2 proficient and deficient contexts. APE2i, PARP1i, ${\mathrm{PoL}}\theta {\mathrm{i}}$ and ATRi refer to the inhibition of APE2, PARP1, ${\mathrm{PoL}}\theta$ and ATR.

Because of the significance of APE2 in DNA repair and genome integrity and the SL phenotype in different genetic backgrounds, it is critical to develop strategies targeting APE2 in monotherapy or combination therapy (1,2,76). Whether APE2 inhibition is globally toxic to cancer cells remains unknown. Genomic and proteomic heterogeneity between cancer types changes the DDR and DNA repair background which in turn may change the effect of APE2 inhibition. Early promising studies have already been conducted concerning APE2 inhibition in an HR-deficient background. Here, we summarize several potential scenarios to target APE2 for chemotherapy in the BRCA1/2-proficient or -deficient contexts (Figure 6).

First, inhibiting or suppressing APE2 functions as a monotherapy of cancers. siRNA-mediated APE2 knockdown leads to substantially more γ-H2AX and micronuclei in pancreatic cancer cell PANC1 under unperturbed conditions (18), suggesting that inhibiting APE2 increased DNA damage in BRCA1/2-proficient cancer cells (Figure 6A). The rising interest in APE2 as a drug target for precision medicine in chemotherapy is largely due to the multiple reports of its SL with BRCA1- and BRCA2-deficient cell lines (10,25,26), suggesting that inhibiting APE2 may lead to the loss of viability in BRCA1/2-deficient cancer cells (Figure 6A). Recently, the first inhibitor of APE2, Celastrol, was identified and characterized (18). Celastrol prevented APE2’s ssDNA binding and 3'-5' exonuclease activity, and attenuated ATR activation in response to DNA damage in pancreatic cancer cells (18). It will be important to test whether inhibiting APE2 function via pharmacological inhibitor Celastrol can be used as a monotherapy of BRCA1/2-proficient or -deficient cancers.

Second, inhibiting APE2 may be combined with PARP1 inhibition for cancer chemotherapy (Figure 6B). Although PARP1i has been developed especially in BRCA1/2-deficient cancers, PARP1i resistance develops quickly in many patients (77,78). It is well established that the cause of PARP1i SL is not due to the inhibition of PARP1’s activity, but instead the phenomena of ‘PARP1 trapping’ in which PARP1 forms a 3’-DNA-protein adduct which prevents replication and transcription (64,79). The kinetics of PARP1 trapping have been proposed to be far more dynamic than previously thought as opposed to forming stable 3'-protein adducts, however stable PARP1-DNA adducts are observed when PARP1 NAD^+^ residues are mutated (80). Despite this, PARP1 still effectively forms 3'-blocked ends even though many PARP1 molecules are moving into and out of the site of DNA damage. APE2 has been shown to effectively process 3'-DNA-protein adducts, thus the rationale for APE2i use in BRCA1- and BRCA2-deficient tumors could be similar to that of PARP1i but less toxicities. Thus, future studies may test whether inhibiting APE2 will overcome PARP1i-resistance in BRCA1/2-proficient or -deficient backgrounds or lead to sensitization to PARP1i (76).

Third, inhibiting APE2 may be combined with Polymerase theta inhibition (Figure 6C). MMEJ proteins such as Pol theta show SL phenotype with BRCA1- and BRCA2-deficiency (25). A study proposes a major role of APE2 in MMEJ and conjectures that the SL phenotype is likely due to APE2’s role in MMEJ rather than its 3'-DNA-protein adduct processing function or its role in BER (26). This may be further queried by testing if APE2 inhibition shows compounding effects with Pol theta inhibition. Roles outside of MMEJ are known for both proteins and combination therapy may increase toxicity in a BRCA1/2 deficient background. As noted previously (Figure 1), APE2 inhibition may offer differential benefit based on its oncogene or tumor suppressor role in different cancer types. Follow up studies will be needed to definitively show what role or roles APE2 performs in cancer cells confer SL phenotype and can be exploited in chemotherapy.

Finally, inhibiting APE2 may be combined with ATR inhibition for cancer therapy (Figure 6D). APE2-knockout has been shown to cause ATR inhibitor sensitivity in multiple cell lines, suggesting that APE2 can possibly confer ATR inhibitor resistance through an unknown mechanism (81,82). Little is known about the role of APE2 in ATR activation under unperturbed conditions, however APE2 is a critical upstream regulator of ATR activation from SSB in *Xenopus* egg extracts and in pancreatic cancer cells (16–18). Replisome screening has also shown that APE2 likely localizes to replication forks, thus APE2 may be critical for replication stress in the absence of ATR activity through an unknown mechanism (58). Increased rates of proliferation and oxidative stress in cancer cells suggest that combination therapy of APE2i and ATRi may be effective against cancer cells without high toxicity in normal tissue. This phenotype is likely due to the roles of APE2 outside of ATR signaling such as 3'-block removal and MMEJ. In addition, ATR inhibition has been shown to cause depletion of HR factors in cancer cell lines (83). Inhibition of APE2 may cause a similar effect due to its proposed upstream regulation of ATR. Chemotherapy targeting APE2 may thus provide a compounding effect of inducing HR deficiency in cancer cells previously proficient in HR, thus rendering them sensitive to PARP1i, Pol Theta inhibitors, and potentially to APE2 inhibition itself. Regardless of if APE2i can itself induce HR cofactor depletion, APE2i combination therapy with ATRi poses an exciting new direction for chemotherapy development due to their synergistic quality.

In addition to being a chemotherapy target, APE2 may contribute to toxicity experienced by patients receiving cisplatin chemotherapy. Mice exposed to cisplatin had dramatic upregulation of APE2 protein in proximal tubule kidney cells (6). Human HK2 cells showed the same phenotype (6). Cisplatin is standard chemotherapy for solid tumors and is generally effective, however standard levels of cisplatin chemotherapy often induce acute kidney injury (AKI) (84). After cisplatin exposure, APE2 abundance is upregulated and more APE2 localizes in the mitochondria (6). In the mitochondria, APE2 localizes with Myosin Heavy Chain-9 (MYH9) which is associated with nephritis (6). Hu et al found that APE2 knockout mice did not show AKI after cisplatin exposure (6). Whether APE2 inhibition also attenuates AKI after cisplatin exposure is not known, however the clinical significance of preventing AKI and allowing high dose cisplatin chemotherapy through APE2 inhibition or targeted immunotherapy presents an exciting prospect.

## PERSPECTIVE ON FUTURE STUDIES OF APE2 IN CANCER

Although studies have started to elucidate APE2’s enzymatic activities and roles in genome and epigenome integrity (1,2), many questions remain unanswered with regard to the mechanisms of APE2 in the context of cancer.

First, it is critical is to determine whether APE2 is a driver and/or passenger of cancer. Although several studies have shown the upregulation of APE2 in tumor tissues compared with normal tissues (27–29), it remains unknown whether APE2 overexpression directly leads to cancer development. APE2 gene is often found mutated (27); however, it is unclear whether and what mutations within APE2 gene is related to cancer development. To understand how APE2’s function in cancer differs from its function in normal tissues, a more comprehensive body of work is needed on its catalytic function and regulation. Some tissue-specific cancer models in genetically modified mice may help to answer these questions.

Second, identifying and developing APE2-specific inhibitors for its roles in DNA repair and immune responses are urgently needed. Due to APE2’s SL phenotype with deficiencies in BRCA1, BRCA2, TDP1, or RNase H2 (10,25), it is critical to screen and develop APE2-specific inhibitors from libraries of small molecule compounds. A small molecular inhibitor of APE2, Celastrol, has been characterized as an inhibitor of APE2’s ssDNA interaction and 3'-5' exonuclease (18). Pancreatic cancer cell sensitivity to Celastrol is encouraging; however, follow-up studies using mouse cancer models with different genetic backgrounds (e.g. BRCA1/2-deficiency) will reveal the implication of targeting APE2 functions via Celastrol in more a pre-clinical setting. In addition, it remains unexplored if APE2 inhibition causes toxicity in normal tissue types as opposed to cancer tissue. Reduced lymphocyte development and immune system impairment is possible considering APE2’s role in these processes. While Celastrol has been characterized as an inhibitor of APE2, it is also known to inhibit other proteins such as HSP90 in cancer cells and MD2 in macrophages (85–87). Precision therapy targeting APE2 will likely require further development of Celastrol derivatives and additional different small molecular inhibitors with limited off target effects.

Third, potential post-translational modifications (PTMs) of APE2 and underlying regulatory mechanisms are currently unknown. Accumulating evidence in bioinformatics and large-scale ‘omics’ analyses have suggested that human APE2 is modified via PTMs including ubiquitination and phosphorylation (88–90). However, more mechanistic studies are needed to demonstrate the exact PTMs on APE2 and to uncover the biological significance of APE2 PTMs. For example, is APE2 ubiquitinated and if so, is APE2 ubiquitination important for APE2 abundance and/or catalytic functions? Is APE2 modified by different PTMs in cancer cells vs. normal cells?

Lastly, can targeting APE2 be combined with immunotherapy? Inhibiting DDR pathways such as ATR, ATM, and PARP1 can induce innate and adaptive immune responses alone or in combination with chemo-radiotherapy and/or immunotherapy (91). It remains to be determined whether APE2 regulates innate/adaptive immune response and whether targeting APE2 functions in B cells can be combined with immunotherapy approaches for cancer therapeutics.

Taken together, in this survey and summary, we have provided the current understanding of functions and regulatory mechanisms of APE2 in immune response, DNA damage response, and DNA repair in the cancer context. We also summarize strategies and future perspectives on targeting APE2 alone and/or in combination with other therapeutic approaches in precision medicine.
